# Supplementary material for: The Chymase Mouse Mast Cell Protease-4 Regulates Intestinal Cytokine Expression in Mature Adult Mice Infected with Giardia intestinalis
Source: Cells. 2020 Apr 9;9(4):925. doi: 10.3390/cells9040925 (PMC7226739; doi:10.3390/cells9040925)
Supplement: Supplementary file 1 [file cells-09-00925-s001.pdf]

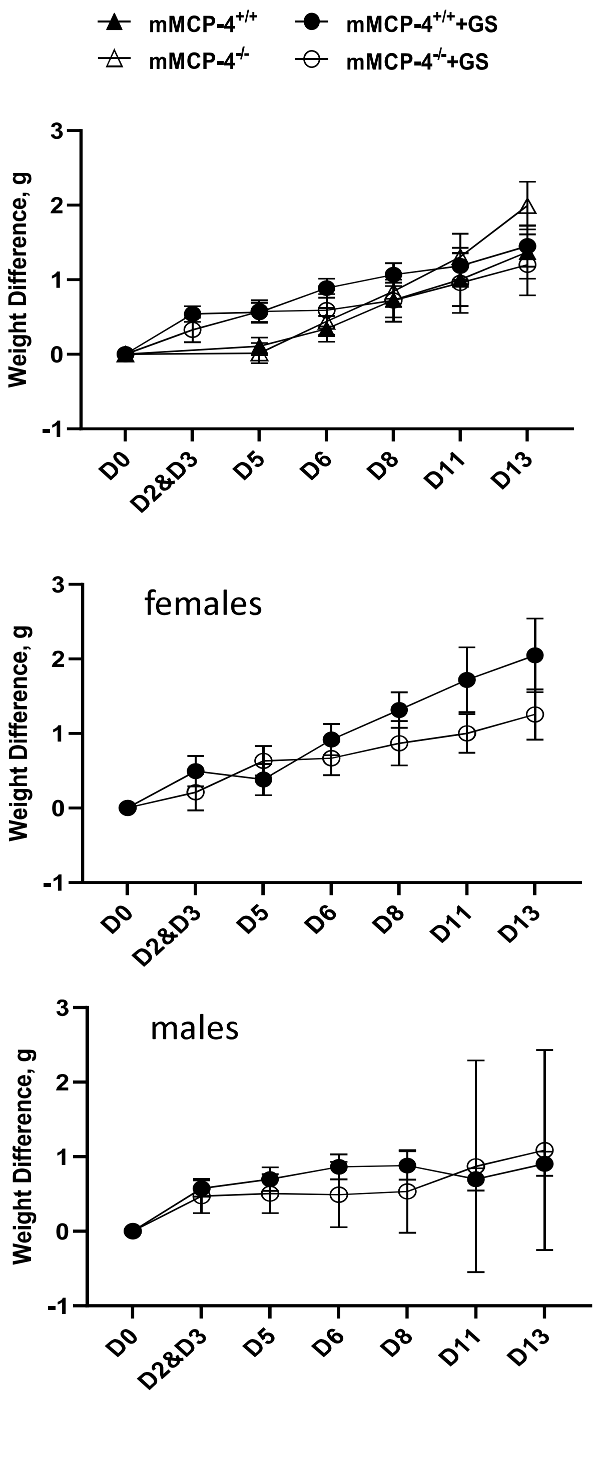

Supplement Figure 1. Weight changes in infected young mMCP-4$^{+/+}$ and mMCP-4$^{-/-}$ mice. Weight changes during 13 days in female and male young (≈10 weeks old) mMCP-4$^{+/+}$ and mMCP-4$^{-/-}$ littermate mice infected with 10^6 *Giardia* GS trophozoites.

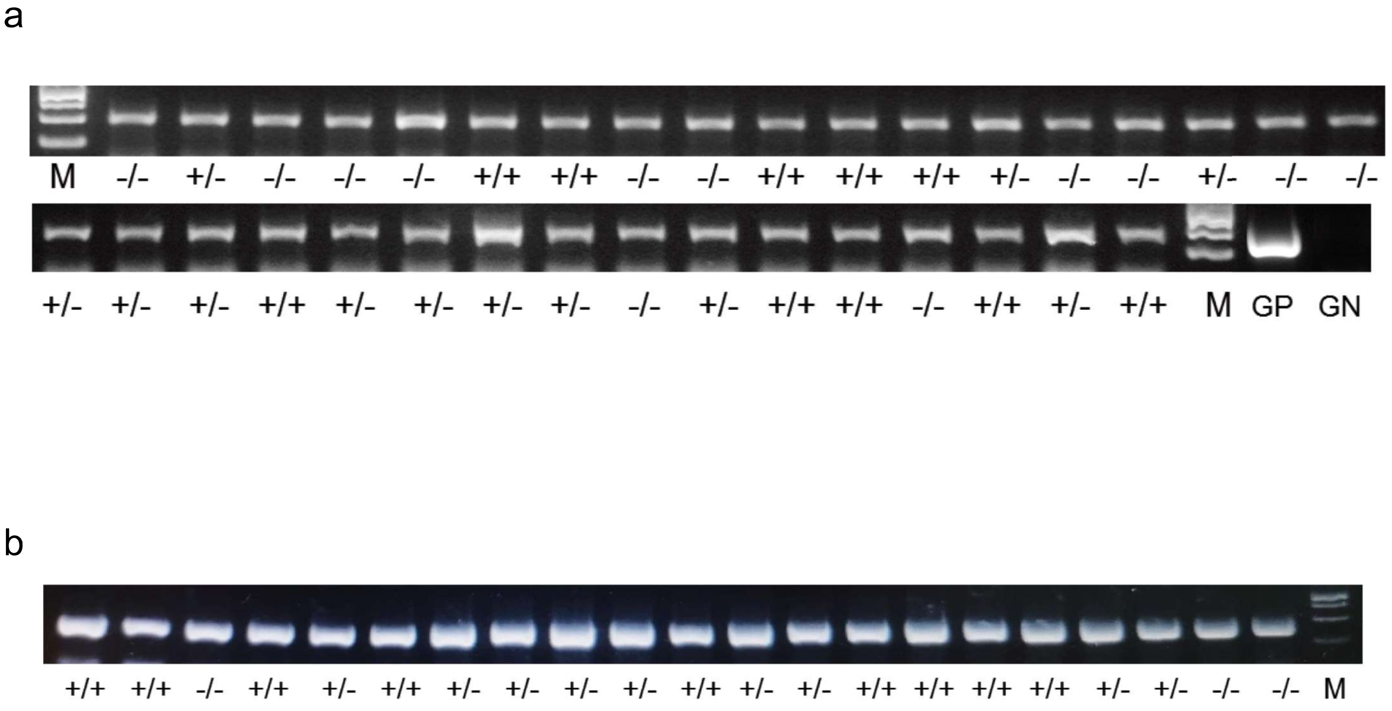

Supplement Figure 2. Identification of *Giardia* by nested PCR in faecal and intestinal samples. a) Faeces collected 13 dpi and b) intestines collected day 8 dpi from the *Giardia*-infected mice**.** +/+ = mMCP-$4^{+/+}$, +/- = mMCP-$4^{+/-}$, -/- = mMCP-$4^{-/-}$ GP = Giardia Postitive, GN = Giardia Negative, M = DNA Ladder
